# Impact of age and comorbidities on real-world outcomes in advanced breast cancer patients treated with palbociclib in first line: a nation-wide Danish retrospective study

**DOI:** 10.2340/1651-226X.2025.43226

**Published:** 2025-06-11

**Authors:** Alan Celik, Laurits Sebastian Dahl, Rasmus Garly, Vesna Glavicic, Maja Bendtsen Sharma, Sophie Yammeni, Humma Khan, Daniel Sloth Hauberg, Hanne Schultz Kapel, Ann Knoop, Tobias Berg

**Affiliations:** a Danish Breast Cancer Group, Rigshospitalet, Copenhagen University Hospital, Copenhagen, Denmark; b Zealand University Hospital, Emergency Department, Nykoebing F, Denmark; c Department of Oncology, Zealand University Hospital, Naestved, Denmark; d Department of Oncology, Aarhus University Hospital, Aarhus, Denmark; e Department of Oncology, Aalborg University Hospital, Aalborg, Denmark; f Pfizer ApS, Ballerup, Denmark; g Department of Clinical Oncology, Rigshospitalet, Copenhagen University Hospital, Copenhagen, Denmark

**Keywords:** Advanced breast cancer, Palbociclib, CDK4/6 inhibitor, survival, age, comorbidity

## Abstract

**Background and purpose:**

Palbociclib, a cyclin-dependent kinase 4/6 (CDK4/6) inhibitor, combined with aromatase inhibitors (AI), can be used in first-line treatment for estrogen receptor (ER) positive, human epidermal growth factor receptor 2 (HER2) normal advanced breast cancer (ABC). This study aims to assess the impact of age and comorbidities on the progression-free survival (PFS) and overall survival (OS) of patients treated with palbociclib and AI.

**Materials and methods:**

This nationwide, retrospective cohort study included 604 women with ER positive, HER2 normal ABC treated with palbociclib and an AI between 2017 and 2021. Data were obtained from the Danish Breast Cancer Group database. Survival outcomes were analyzed based on age, Charlson Comorbidity Index (CCI), number of comorbidities, and comorbidity type. PFS and OS were estimated using the Kaplan–Meier method.

**Results:**

Median PFS for all patients was 30.6 months (95% confidence interval [CI], 27.4–34.2), and median OS was 55.6 months (95% CI, 51.8–58.9). Patients aged 65–75 years had significantly longer PFS and OS (*p* = 0.031 and *p* = 0.012) than patients aged under 65 and over 75 years. Visceral metastases were associated with shorter PFS and OS (*p* = 0.005). Comorbidity burden, including CCI score and comorbidity type, did not significantly affect survival outcomes.

**Interpretation:**

In our data set, univariate analyses suggest age and visceral metastases to be potential factors influencing outcomes in patients with ABC treated with palbociclib plus an AI. Comorbidities, did not significantly impact survival, suggesting that palbociclib is well-tolerated in patients with varying health profiles.

**ClinicalTrials.gov ID:**

NCT06307457.

## Introduction

The treatment landscape for advanced breast cancer (ABC) has drastically evolved with the arrival of targeted therapies, offering new hope for improved outcomes. Among these, selective cyclin-dependent kinase 4/6 (CDK4/6) inhibitors combined with an aromatase inhibitor (AI) have emerged as a first-line standard of care for estrogen receptor (ER) positive, human epidermal growth factor receptor 2 (HER2) normal ABC. This treatment has shown substantial efficacy in clinical trials, markedly extending progression-free survival (PFS) and overall survival (OS) [1–6]. However, the real-world effectiveness of this regimen can be influenced by various patient-specific factors, notably age and the presence of comorbidities. Older patients or patients with high comorbidity burden are often not included in trials given strict inclusion/exclusion criteria.

Understanding the interplay between patient characteristics and treatment outcomes is crucial for optimizing therapy and personalizing care in ABC. Ageing organ function can impact pharmacokinetics, treatment tolerance, and patient outcomes. Similarly, comorbidities, which are more common in the aging population, may complicate cancer treatment due to potential drug interactions and alteration of drug effectiveness. Evaluating how these factors affect the effectiveness of palbociclib in combination with an AI is essential to refining treatment protocols and ensuring reasonable care across diverse patient populations.

Several real-world studies have evaluated palbociclib, both with and without an AI, in older patients and those with comorbidities, with only two studies formally reviewing impact of comorbidities on outcomes [7–12]. However, such data have not yet been explored in a Danish setting. This study aims to explore the impact of age and comorbidities on treatment outcomes of first-line therapy with the CDK4/6 inhibitor palbociclib in combination with an AI in ER-positive, HER2-normal ABC. This study builds on a previously published cohort, extending follow-up and focusing exclusively on first-line treatment with palbociclib combined with an AI [13].

## Material and methods

### Study design

This is a nationwide, retrospective cohort study involving all departments of oncology in Denmark.

### Patient selection

All known women aged 18 years or above diagnosed with primary (*de novo*) or recurrent ABC were included. All patients included had ER positive, HER2 normal disease and initiated first-line treatment with palbociclib in combination with an AI between January 1^st^, 2017, and December 31^st^, 2021. ER and HER2 status were based on metastatic tissue or – if not available – on primary tumor tissue.

### Data source

The Danish Breast Cancer Group (DBCG) database was utilized in this study to gather information on date of primary diagnosis, date of ABC diagnosis, treatment ((neo)adjuvant and for advanced disease), date of progression (based on imaging and/or clinical examination), reason for treatment discontinuation and location of metastases. Information regarding comorbidities was gathered from the Danish National Patient Registry. Information concerning vital status was obtained from the Danish Civil Registration System.

### Study endpoints

Primary endpoints were PFS and OS for all patients.

Secondary endpoints were PFS and OS stratified by age (< 65, 65–75 and ≥ 75 years), comorbidities (severity, number, and type), type of metastases (visceral and bone-only disease), disease presentation (*de novo* metastatic or recurrent), and endocrine resistance. *De novo* metastatic disease was defined as distant metastases at diagnosis or within 90 days of (neo) adjuvant therapy initiation. Endocrine resistance was defined as recurrent advanced disease within 12 months of completing or during adjuvant endocrine therapy (ET); endocrine sensitivity was defined as recurrent advanced disease after 12 months of completing adjuvant ET; recurrent patients having not received adjuvant ET and patients with *de novo* ABC were also counted as sensitive.

### Measures

Comorbidity-subgroup analyses were split into three: severity, number of comorbidities and type at ABC diagnosis. The Charlson Comorbidity Index (CCI) was used to score severity including diagnoses made within the 10 years preceding the ABC diagnosis [14]. Patients were subdivided into four groups based on their CCI-score (0, 1, 2, and 3+). Patients were further divided into three comorbidity groups based on number of comorbidities (0, 1, and 2+ comorbidities). Splitting the subgroup analysis according to number of comorbidities per patient therefore puts equal weight on all types of comorbidities. Patients were also subdivided based on type of comorbidity into three main groups: cardiac, vascular, and metabolic diseases, following the diseases used in the CCI.

### Statistical analysis

PFS was defined as the time from date of ABC diagnosis to either progression or death of any cause, whichever occurred first. OS was defined as the time from the date of ABC diagnosis to death from any cause. Patients were followed up until February 1^st^, 2024, and were censored at this point in time, if progression-free and/or alive. Estimates were calculated using the Kaplan–Meier method. Differences in both, PFS and OS across subgroups, were assessed using the Log-Rank method (univariate analysis). As this was a descriptive, exploratory study, we focused on univariate analyses. This approach was chosen to allow a clear and direct interpretation of real-world patterns without the added complexity or assumptions of multivariable modeling. Follow-up duration was calculated using the reverse Kaplan–Meier method. PFS, OS and follow-up duration were reported as medians with confidence intervals (CIs).

## Results

### Study population

**Figure 1:**
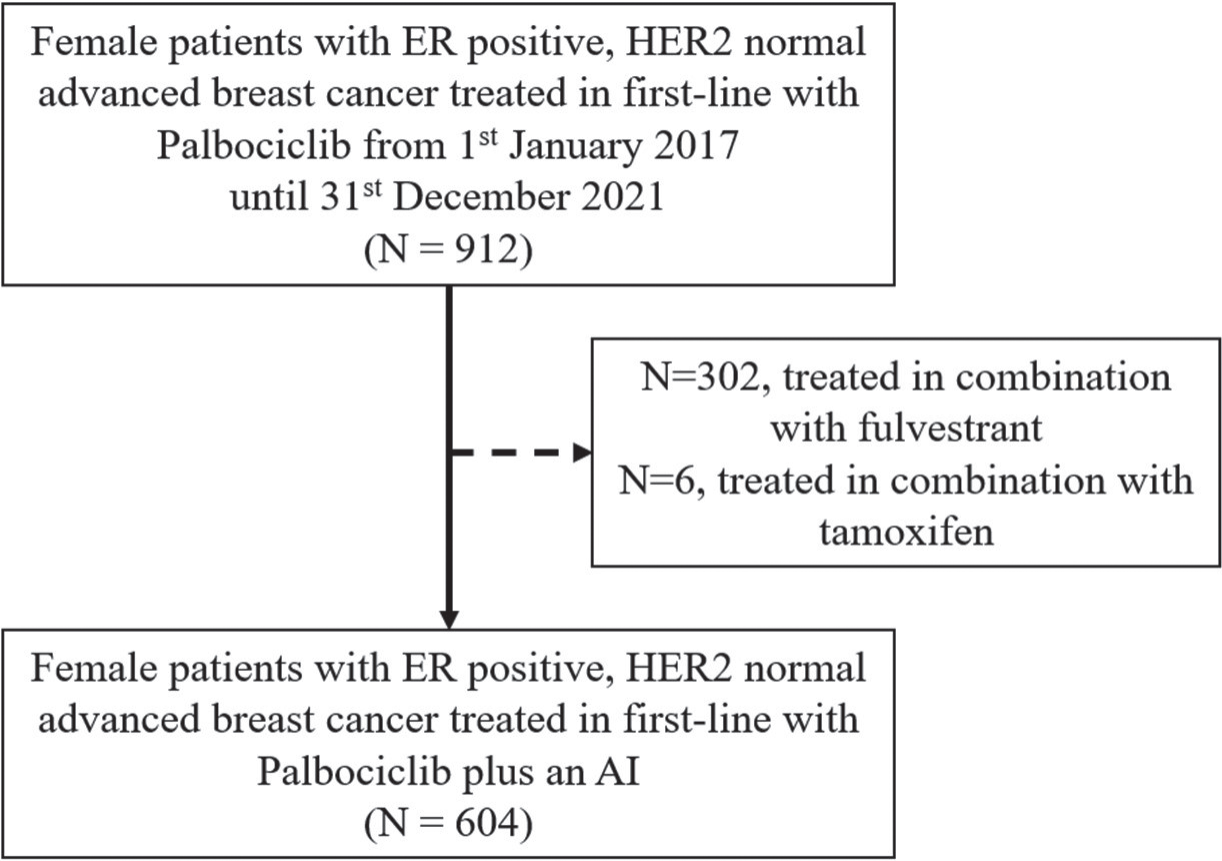
Flowchart of patient inclusion. ER: estrogen receptor; HER2: human epidermal growth factor receptor 2; AI: aromatase inhibitor.

During the study period, 604 patients received palbociclib in combination with an AI as first-line therapy across all departments of oncology in Denmark (Figure 1). Patient characteristics are presented in Table 1.

**Table 1:** Patient characteristics.

Patients	*N* = 604
**Median age**	68.9 (32–91)
Under 65 years	212 (35%)
65–75 years	243 (40%)
Over 75 years	149 (25%)
**CCI**	
0	400 (66%)
1	101 (17%)
2	45 (7.5%)
3+	58 (9.6%)
**Number of comorbidities**	
0	400 (66%)
1	147 (24%)
2+	57 (9.4%)
**Type of comorbidity**	
Cardiac	23 (3.8%)
Vascular	45 (7.5%)
Metabolic	45 (7.5%)
**Sites of cancer**	
Visceral	321 (53%)
Bone-only	145 (24%)
**Endocrine resistance**	
Endocrine resistant	67 (11%)
Endocrine sensitive	537 (89%)
***De novo* metastatic disease**	198 (33%)

CCI: Charlson comorbidity index.

Number of patients displayed as *N* (%).

Median age was 68.9 years at baseline. A total of 66% (*n* = 400) of the patients had a CCI of 0, 17% (*n* = 101) a CCI of 1, 7.5% (*n* = 45) a CCI of 2, and 9.6% (*n* = 58) a CCI of 3 or more. In terms of the number of comorbidities, 147 patients (24%) had one comorbidity and 57 (9.4%) had two or more comorbidities at baseline. Both vascular and metabolic disease were present in 45 patients (7.5%) and 23 patients (3.8%) presented with cardiac disease. Most patients had visceral metastases, accounting for 321 patients (53%), while 145 (24%) had bone-only metastases. The remaining patients (23%) presented with metastases to distant lymph nodes, skin, muscle, or the contralateral breast, with or without concurrent bone metastases. Endocrine resistance was observed in 67 patients (11%), and 537 (89%) were classified as endocrine sensitive. Additionally, 198 patients (33%) presented with *de novo* metastatic disease.

### Outcomes

For the overall 604 patients, median PFS was 30.6 months (95% CI, 27.4–34.2), and median OS was 55.6 months (95% CI, 51.8–58.9). A total of 322 patients died during the study period, and 430 patients had an event in form of progression or death during the study period. Kaplan–Meier graphs for PFS and OS are included in Supplementary Figure 1. Median follow-up for PFS and OS were 67.4 months (95% CI, 62.9–69.9) and 66.9 months (95% CI, 63.4–69.6), respectively.

Based on univariate analysis, age-groups showed a significant difference in terms of PFS and OS, with patients aged 65–75 years having a the longest median PFS and OS (*p* = 0.031 and *p* = 0.012). Patients with visceral metastases had a shorter median PFS and OS compared to patients without visceral involvement (*p* = 0.005 and *p* = 0.005). Endocrine-sensitive patients had a longer median PFS and OS compared to endocrine-resistant patients (*p* = 0.047 and *p* = 0.041). The median PFS in patients with de novo metastatic disease was 32.0 months (95% CI: 25.5–43.3) and median OS was 58.9 months (95% CI: 52.6–70.3).

In this univariate analysis, CCI did not significantly affect outcomes in terms of PFS and OS when stratified based on score (*p* = 0.061 and *p* = 0.080). Similarly, the number of comorbidities did not significantly affect PFS or OS (*p* = 0.2 and *p* = 0.093). Specific comorbidity subgroups, including cardiac, vascular, and metabolic diseases, did not show significant differences in outcomes.

All outcomes are summarized in Table 2.

**Table 2:** Outcomes in terms of median real-world progression free survival and overall survival.

Characteristic	*N*	Median (95% CI) progression free survival	*p* ^1^	Median (95% CI) overall survival	*p* ^1^
**All patients**	604	30.6 (27.4, 34.2)		55.6 (51.8, 58.9)	
**Age group**			0.031		0.012
Under 65 years	212	25.8 (22.2, 30.7)		52.6 (48.6, 59.2)	
65–75 years	243	35.7 (30.4, 42.2)		60.8 (55.6, 78.3)	
Over 75 years	149	29.8 (23.6, 37.2)		50.1 (41.4, 58.6)	
**Visceral metastases**			0.005		0.005
Non-Visceral	283	34.6 (31.1, 39.8)		59.2 (56.6, 69.8)	
Visceral	321	24.2 (19.8, 30.3)		50.6 (44.0, 55.7)	
**Bone-only**			0.14		0.2
Bone-only	144	34.6 (27.7, 42.6)		58.3 (51.8, NA)	
Non-bone-only	460	29.0 (25.4, 33.1)		54.2 (50.1, 58.6)	
**Endocrine Status**			0.047		0.041
Endocrine resistant	67	21.7 (13.8, 37.2)		43.5 (32.2, 59.2)	
Endocrine sensitive	537	31.8 (28.5, 34.6)		56.6 (52.6, 60.8)	
**De novo metastatic**	198	32.0 (25.5, 43.3)		58.9 (52.6, 70.3)	
**Charlson Comorbidity Index**			0.061		0.080
0	400	32.8 (28.3, 36.7)		57.6 (53.9, 66.7)	
1	101	32.0 (26.0, 45.8)		50.1 (43.6, 58.9)	
2	45	29.7 (17.1, 38.3)		53.1 (43.3, NA)	
3+	58	21.5 (18.0, 33.2)		45.2 (37.7, 58.3)	
**Number of comorbidities**			0.2		0.093
0	400	32.8 (28.3, 36.7)		57.6 (53.9, 66.7)	
1	147	27.9 (23.8, 37.9)		50.1 (44.5, 58.0)	
2+	57	23.6 (19.3, 35.6)		52.7 (41.2, NA)	
**Type of comorbidity**					
***Cardiac***			> 0.9		0.7
No	581	30.7 (27.3, 34.3)		55.6 (51.8, 58.9)	
Yes	23	27.9 (22.2, NA)		61.2 (45.6, NA)	
***Vascular***			0.9		0.3
No	559	30.3 (27.0, 34.2)		56.1 (52.4, 59.2)	
Yes	45	32.3 (22.9, 44.9)		47.4 (42.9, NA)	
***Metabolic***			0.4		0.7
No	559	31.1 (27.7, 34.6)		56.1 (52.4, 59.2)	
Yes	45	25.5 (17.7, 36.5)		50.1 (39.8, NA)	

1 Log-Rank (univariate analysis).

## Discussion

Our study aimed to explore the impact of age and comorbidities on the treatment outcomes of first-line therapy with palbociclib and an AI in ER positive, HER2 normal ABC.

In our data set, univariate analyses suggest age as a significant factor influencing both PFS and OS. The superior outcomes in the 65–75 age group could be due to several factors, including differences in tumor biology and burden, treatment tolerance, and adherence to therapy. While older patients (above 75 years) showed a PFS similar to younger patients (29.8 months, 95% CI, 23.6–37.2), their OS was notably not surprisingly shorter (50.1 months, 95% CI, 41.4–58.6) [15]. In a recent study, in patients aged 75 years or older the point estimate of PFS was lower compared to the one found in our study (20.5 months (95% CI, 17.5–27.3)) with a median OS of 47.8 months (95% CI, 40.7–NA) [16].

The presence of visceral metastases was associated with significantly worse outcomes (*p* = 0.005 for both PFS and OS). These findings align with prior findings suggesting that visceral involvement is a marker of more aggressive disease [13]. Furthermore, we found that patients with endocrine sensitive disease experienced significantly improved outcomes compared to those with resistant disease (*p* = 0.047 and *p* = 0.041, for PFS and OS respectively).

Interestingly, the severity of comorbidities, assessed through the CCI, and the number of comorbidities did not statistically significantly impact PFS or OS, but did however show a numerical trend favoring a CCI score of 0. Similarly, the number of comorbidities did not significantly affect survival outcomes (*p* = 0.093). Moreover, specific comorbid conditions, such as cardiac, vascular, and metabolic diseases, were not associated with significant differences in survival outcomes (*p* > 0.05 for all comparisons); however, sample sizes were small in these subgroups.

The lack of an impact of comorbidities on PFS and OS in our dataset might suggests that palbociclib in combination with an AI is a viable therapeutic option across a broad range of patient profiles, including those with multiple comorbidities or severe comorbidity burden. This is crucial in clinical practice, especially for older patients, who are more likely to present with comorbid conditions. ESMO-MCBS scores differ among the available three CDK4/6 inhibitors, with ribociclib receiving the most favorable ratings, though it is important to acknowledge that no specific CDK 4/6 inhibitor is recommended in first-line in the ESMO/ESO-guidelines [6, 17]. Additionally, ribociclib has been associated with a higher incidence of clinically significant (Class C/D) drug–drug interactions (especially regarding QT interval prolongation) compared to palbociclib, a concern particularly pertinent in older patients who often manage multiple comorbidities and medications [18]. No head-to-head comparisons between the three approved CDK 4/6 inhibitors have been made yet concerning outcomes. As this study focused on the use of palbociclib, we are unable to make any comparisons or conclusions about other available CDK4/6 inhibitors with respect to the outcomes stratified by comorbidities.

This study has several strengths and limitations. By utilizing the DBCG national database, we ensured that all known women in Denmark treated with palbociclib plus AI during the study period were included, minimizing geographical and socioeconomic bias. No information regarding performance status, quality of life during treatment, and data concerning the safety of treatment was available. In addition, due to the retrospective nature of our study, missingness cannot be excluded. The small sample sizes of some subgroups also presented a limitation. Finally, a key limitation of the study is the lack of multivariable analysis, which restricts our ability to assess potential confounding factors. However, given the descriptive aim of the study, we prioritized an unadjusted approach to capture clinically observable trends in a real-world setting. We recognize that future studies with larger and more uniform cohorts may benefit from multivariable analyses to further clarify these relationships. Accordingly, further assessments, including prospective validation of our findings are warranted.

## Conclusion

In conclusion, our findings suggest that palbociclib in combination with an AI is an effective treatment across different patient subgroups in ABC, though certain groups may derive a greater benefit than others. Our real-world univariate analyses showed that patients aged 65–75 years, patients without visceral metastases, and endocrine-sensitive patients had the greatest survival benefit. Comorbidities did not significantly impact outcomes, indicating that palbociclib-based therapy may be well-tolerated across diverse health profiles. Imbalances between prognostic factors, such as age and comorbidity groups, may have influenced this analysis, highlighting the need for further investigation, including prospective studies.

## Supplementary Material



## Data Availability

All data are stored in the DBCG database. The dataset can be made available to qualified researchers through application to the Danish Breast Cancer Group. Please contact dbcg.rigshospitalet@regionh.dk.
