# Supplementary material for: Impact of age and comorbidities on real-world outcomes in advanced breast cancer patients treated with palbociclib in first line: a nation-wide Danish retrospective study
Source: Acta Oncol. 2025 Jun 11;64:43226. doi: 10.2340/1651-226X.2025.43226 (PMC12175173; doi:10.2340/1651-226X.2025.43226)
Supplement: Supplementary file 1 [file AO-64-43226-s1.pdf]

Supplementary material has been published as submitted. It has not been copyedited, or typeset by Acta Oncologica

Supplementary figure 1: Kaplan-Meier plots of (A) progression-free and (B) overall survival

A

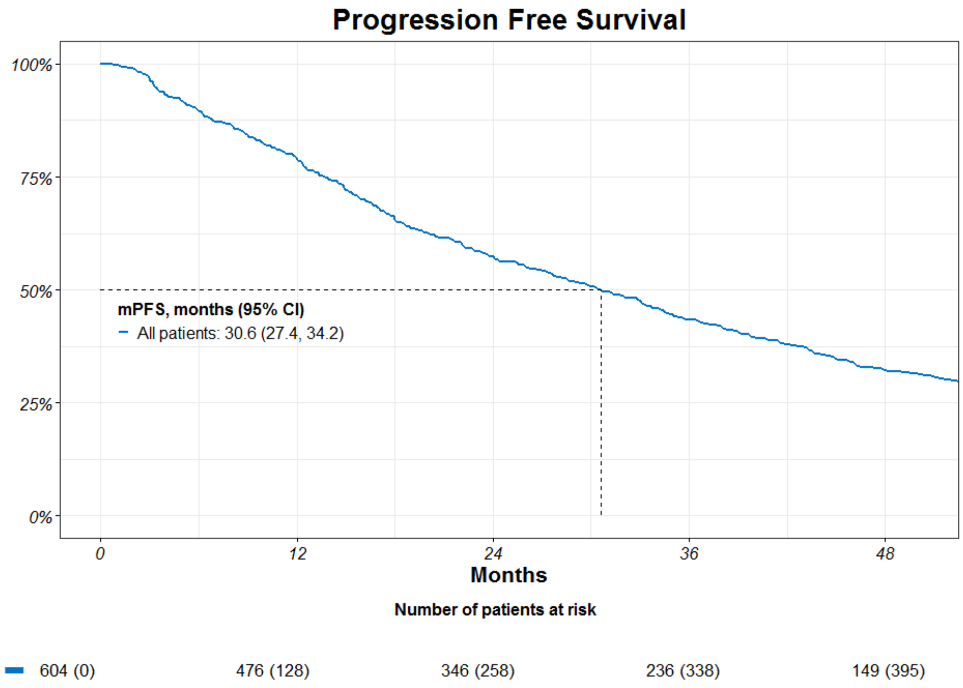

B

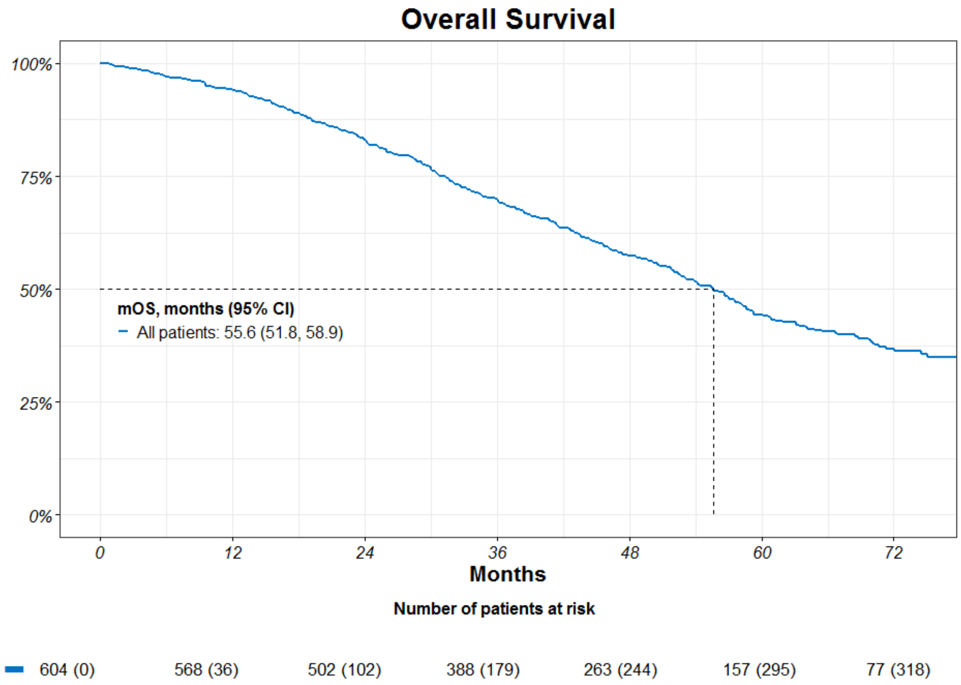

mPFS: median progression-free survival, mOS: median overall survival, CI: confidence interval
